# Distinct N-Linked Immunoglobulin G Glycosylation Patterns Are Associated With Chronic Pathology and Asymptomatic Infections in Human Lymphatic Filariasis

**DOI:** 10.3389/fimmu.2022.790895

**Published:** 2022-03-25

**Authors:** Tomabu Adjobimey, Achim Hoerauf

**Affiliations:** ^1^ Institute of Medical Microbiology, Immunology and Parasitology Institut for Medical Microbiology, Immunology and Parasitology (IMMIP), University Hospital Bonn, Bonn, Germany; ^2^ Faculté des Sciences et Techniques (FAST), Université d’Abomey Calavi, Abomey Calavi, Benin; ^3^ Bonn-Cologne Site, German Center for Infectious Disease Research Deutsches Zentrum für Infektionsforschung (DZIF), Bonn, Germany

**Keywords:** IgG, filariasis, UPLC-FLD/ESI-MS, N-glycosylation, galactosylation, fucosylation, sialylation, GlcNac

## Abstract

Lymphatic filariasis presents a complex spectrum of clinical manifestations ranging from asymptomatic microfilariaemic (MF+) to chronic pathology (CP), including lymphedema and elephantiasis. Emerging evidence suggests a link between the physiopathology of filarial infections and antibody properties. Post-translational glycosylation has been shown to play a key role in the modulation of antibodies’ effector functions. Here, we investigated the link between total IgG-N-glycosylation patterns and the physiopathology of human lymphatic filariasis using UPLC-FLD/ESI-MS comparison of N-glycan profiles of total IgG purified from endemic normals (EN), MF+, and CP patients. We detected a total of 19 glycans released from all IgG samples. Strikingly, agalactosylated glycan residues were more prominent in EN, whereas sialylation and bisecting GlcNac correlated with asymptomatic infections. While IgG from all three clinical groups expressed high levels of fucosylated residues, significantly lower expressions of afucosylated IgG were seen in MF+ individuals compared to EN and CP. Our data reveal distinct N-linked IgG glycan profiles in EN, MF+, and CP and suggest that IgG galactosylation and sialylation are associated with chronic pathology, whereas agalactosylation correlates with putative immunity. The results also indicate a role for sialylation, fucosylation, and bisecting GlcNac in immune tolerance to the parasite. These findings highlight the link between N-glycosylation and the physiopathology of lymphatic filariasis and open new research avenues for next-generation therapeutic formulations against infectious diseases.

## Introduction

Lymphatic filariasis, commonly known as elephantiasis, is a neglected tropical disease caused by vector-borne filarial parasites *Wuchereria bancrofti, Brugia malayi*, and *Brugia timori* (1). Recent estimations of the WHO indicate that 859 million people in 50 countries live in areas requiring chemotherapy to stop the spread of the infection. The global baseline estimate of people affected by lymphatic filariasis is 25 million men with hydrocele and over 15 million people with lymphoedema (2). In endemic areas, most infected individuals remain asymptomatic, showing no external signs of the infection while bearing microfilaria (MF+) and contributing to the transmission of the parasite (2). In contrast, putatively immune individuals, also known as endemic normals (EN), remain infection-free despite continuous exposition to the vector (3). In some of the exposed individuals, the infection develops into chronic pathology (CP), manifesting as lymphoedema (tissue swelling), elephantiasis (skin/tissue thickening) of limbs, or hydrocele (scrotal swelling). Besides morbidity and its economic drawbacks, the disfiguring sequels of LF often lead to social stigma and considerable psychological burden (4). The clinical outcomes of the infection are tightly linked to the host’s immune reactivity. Typically, alongside classical parasite-induced Th2 immune responses, asymptomatic patients present a strong immune-regulated profile with high levels of regulatory cells, anti-inflammatory cytokines. This immune-regulated environment is associated with elevated levels of the non-cytolytic antibody IgG4 (3, 5, 6). It is well established that antibodies play a key role in protection and pathology across infectious diseases. While the antibody variable domain facilitates antibody binding to antigens and the blockade of infection, the constant, crystallizable fragment (Fc) mediates cross-talk with the innate immune system. The biological activity of the Fc region is controlled genetically *via* class switch recombination, resulting in the selection of distinct antibody isotypes and subclasses (7). In helminth infections, the IgG4 subclass was seen to be associated with tolerance of the parasite, whereas IgG1 and IgG3 are associated with parasite clearance and or pathology (5). New pieces of evidence suggest a second level of control *via* post-translational changes in antibody glycosylation. Fc glycans were shown to be critical for maintaining both the proinflammatory and the anti-inflammatory effector functions of IgG (8). Indeed N-glycan moieties of IgG-Fc were shown to significantly impact antibody stability and effector functions (7, 9). All four human IgG subclasses exhibit two variable bi-antennary glycans in their Fc region, attached at the conserved Asn297 site (10). The Fc glycans in the IgG molecule are bi-antennary glycans with varying fucose content, bisecting *N*-acetylglucosamine (GlcNac), galactose (Gal), and sialic acid; most IgG molecules are fucosylated. These glycan structures have profound impacts on antibody functions and thereby on health. Agalactosylated and asialylated IgG glycoforms, for example, were seen to be particularly abundant in chronic inflammatory diseases such as rheumatoid arthritis, systemic lupus erythematosus, inflammatory bowel disease (IBD), HIV, and mycobacterial infections (11–16). Global immune activation studies reveal that IgG1 galactose deficiency correlated with parasitic infections or asthma (17). Conversely, sialylation has been associated with anti-inflammatory properties and has, for example, been seen to reduce pathogenic properties of IgG autoantibodies (18). Recent data from our group have suggested that helminths modulate both the quantity and the function of antibodies secreted in their host (19). This qualitative modulation of antibodies’ properties may have a profound impact on the ability of the host to tolerate or eliminate adult worms and microfilaria. Here we analyzed the link between different antibody-glycosylation patterns and the physiopathology of human lymphatic filariasis using mass spectrometry. Ultimately, this work provides critical new insights into the functional roles of antibody glycosylation and lays initial foundations for leveraging antibody glycosylation to drive prevention and control across diseases.

## Materials and Methods

### Study Population and Ethic Statement

The present study used archived material from previous projects. Patients and endemic controls’ samples were collected between 2009 and 2010 in the Nzema East District in the western region of Ghana endemic for LF. No other human filarial species were endemic in the region. Written informed consent was obtained from all participants. Exclusion criteria included abnormal hepatic and renal enzyme levels (γ-glutamyltransferase > 28 U/L, glutamyl pyruvic transaminase > 30 U/L, creatinine > 1.2 mg/100 mL) assessed by dipstick chemistry, alcohol, drug abuse, or antifilarial therapy in the past 10 months. The participants in the study were examined by a clinician using physical methods and a portable ultrasound machine (180 Plus; SonoSite, Bothell, WA) as described previously (20). Ethical clearance was given by the Committee on Human Research Publication and Ethics at the University of Science and Technology in Kumasi and the Ethics Committee at the University Hospital Bonn. Microfilaremia was determined by microscopic examination of fingerprick night blood samples as published (20). Endemic normals (EN) were defined as residing in the endemic region but free of infection (CFA-, MF-, n=10), clinically asymptomatic microfilaraemic (CFA+, MF+, n=10) chronic pathology patients (CP) with lymphedema or elephantiasis (n=10), negative for filarial antigen. Since IgG-glycosylation patterns can be affected by aging and estrogens levels (21, 22), only middle-aged males (41-48 years) were included in the analysis (**Table 1**). Patients receiving any other medical treatments were excluded from the analysis.

**Table 1:** Clinical characteristics of patients and controls.

*N°*	Age in years	Gender	MF/10 ml	CFA	LE/Hy	Antifilarial antibody titer	Clinical classification
*1*	45	M	0	-	-	-	EN
*2*	48	M	0	–	–	–	EN
*3*	43	M	0	-	-	-	EN
*4*	44	M	0	–	–	–	EN
*5*	42	M	0	-	-	-	EN
*6*	40	M	0	–	–	–	EN
*7*	41	M	0	-	-	-	EN
*8*	43	M	0	–	–	–	EN
*9*	45	M	0	-	-	-	EN
*10*	43	M	0	–	–	–	EN
*11*	47	M	1101	+	-	+	MF+
*12*	46	M	2710	+	–	+	MF+
*13*	44	M	4264	+	-	+	MF+
*14*	41	M	2182	+	–	+	MF+
*15*	44	M	5166	+	-	+	MF+
*16*	46	M	1182	+	–	+	MF+
*17*	40	M	271	+	-	+	MF+
*18*	42	M	3864	+	–	+	MF+
*19*	46	M	354	+	-	+	MF+
*20*	44	M	608	+	–	+	MF+
*21*	48	M	0	-	+	+	LE
*22*	46	M	0	–	+	+	LE
*23*	47	M	0	-	+	+	LE
*24*	43	M	0	–	+	+	LE
*25*	41	M	0	-	+	+	LE
*26*	45	M	0	–	+	+	Hy
*27*	41	M	0	-	+	+	Hy
*28*	46	M	0	–	+	+	Hy
*29*	47	M	0	-	+	+	Hy
*30*	40	M	0	–	+	+	Hy

M, Male; MF, Microfilaria; CFA, Circulating Filarial Antigen; EN, Endemic Normal; LE, Lymphedema; Hy, Hydrocele.

### IgG Isolation by Protein G Agarose Beads

**Figure 1:**
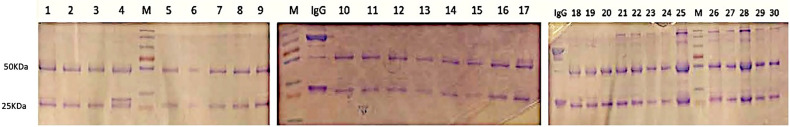
SDS-PAGE analysis of IgG purity from plasma with Protein G resin and acidic elution. Numbers 1-30 indicated the position of the corresponding sample. M, molecular weight marker. SDS-PAGE results to determine the purity of purified IgG showed a distinct band of 50 kDa. Molecular weight marker position corresponds to Ab heavy chain, and the band in 25 kDa position corresponds to IgG light chain.

Complete plasma samples (500μg-1mg) were diluted in 0.4 mL of PBS (Fisher Scientific) and filtered with Amicon ultrafiltration filters (1.5mL, 30 kDa cutoff) at 12.500 r.p.m. (Allegra bench centrifuge) for 3 minutes at 25°C to remove buffer and stabilization media. This washing was repeated twice. Filtered plasma solutions in PBS were incubated with 30μL of protein G beads solutions for 60 minutes for antibody immobilization by IgG-G protein binding. After binding, the solution containing the beads was centrifuged at 12.500 r.p.m. (Allegra bench centrifuge) for 3 minutes at 25°C to remove any unbound excess protein. Centrifugation was repeated three times with 200uL of PBS. Subsequently, the IgG fraction (150uL in PBS) was released from the protein G beads by incubation with 200μL of HCL 100 mM solution (acidic conditions) for 15 minutes. The samples were centrifuged again, and the supernatant containing the IgG was recovered. Each purified IgG solution was neutralized with 40μL of a NaOH 500 mM solution, and the resulting solutions were analyzed by SDS-PAGE for assessing the purification yield (**Figure 1**). IgG samples were then used directly for the next deglycosylation step.

### Deglycosylation and Glycan Isolation

IgG samples (25μg-200μg) were diluted in 300μL ultrapure water and filtered with Amicon^®^ Ultra 0.5mL Centrifugal filters (10,000 NMWL) at 4,500 r.p.m on an AllegraTM X-22R Benchtop Centrifuge for 12 minutes at 25°C to remove metabolites from media. The protein samples were concentrated to 150μL and immediately denatured using 150μL of 1% SDS and under heating at 60°C for 30 minutes. The solutions of denatured IgG were diluted with 350μL of deionized water and concentrated, using Amicon^®^ Ultra 0.5mL Centrifugal filters as described above to remove SDS. The concentrated/denatured protein solutions were treated with 4μL of PNGase F and incubated for 90 minutes at 60°C. After deglycosylation, released N-glycans were isolated from the protein fraction by filtration using Amicon Ultra-0.5 mL Centrifugal filters (10,000 NMWL) at 12,500 r.p.m. (AllegraTM X-22R Benchtop Centrifuge) for 5 minutes at 25°C. The filtrate solutions containing released N-glycans were lyophilized on a TELSTAR LyoQuest Plus -55 freeze dryer (TELSTAR, Terrassa, Spain). The retained fractions were recovered by inverting the filter and transferred by centrifugation (at 1,500 r.p.m. for 1 minute at 25°C) to a second receiver vial and stored at -20°C.

### Procainamide Labeling

Recent reports on LC-FLR/MS reveal glycans labeling with procainamide (4-amino-*N*- (2-diethylaminoethyl) benzamide) as an effective method to improve MS ionization efficiency without compromising LC separation (23). A procainamide labeling solution was prepared following the Waters Application Note. UPLC/FLR/QTof (MS Analysis of Procainamide-Labeled N-glycans). Briefly, 12mg of procainamide and 6mg of sodium cyanoborohydride were dissolved in 0.5mL of a dimethyl sulfoxide (DMSO)/Acetic acid (7/3) mixture. The lyophilized N-glycan samples were reconstituted in 10μL of ultrapure water. The obtained solutions were treated with 50 μL of procainamide labeling solution at 65°C for 3 hours under gentle shaking on a Thermomixer (Eppendorf). After labeling, the labeling solutions were diluted with 200μL Acetonitrile and directly analyzed by UPLC-FLD/ESI-MS under standard conditions (Waters, Application Note. UPLC/FLR/QTof MS Analysis of Procainamide-Labeled N- glycans).

### UPLC-FLD/MS Analysis of Procainamide Labeled N-Glycans

Profiles of procainamide-labeled fluorescent N-glycans from isolated IgG samples were obtained by HILIC UPLC-FLD/ESI-MS. Labeled glycans were separated by UPLC on a HILIC (hydrophilic interaction liquid chromatography) column, and labeled glycans were detected by online fluorescence and ESI-TOF mass detection. Fluorescence detection allowed us to quantify the uniformly labeled glycans peaks with very high sensitivity. In contrast, mass detection was employed to provide structural data for the assignment of peaks to glycan structures based on mass. For quantification, peaks areas were integrated and converted into relative percentages of the total glycan amount defined as the sum of all peaks and normalized to 100%. The UPLC analysis was performed following the standard procedures from Waters (Application Note. UPLC/FLR/QTof MS Analysis of Procainamide-Labeled N-glycans).

### Peak Assignment - Methodology

**Figure 2:**
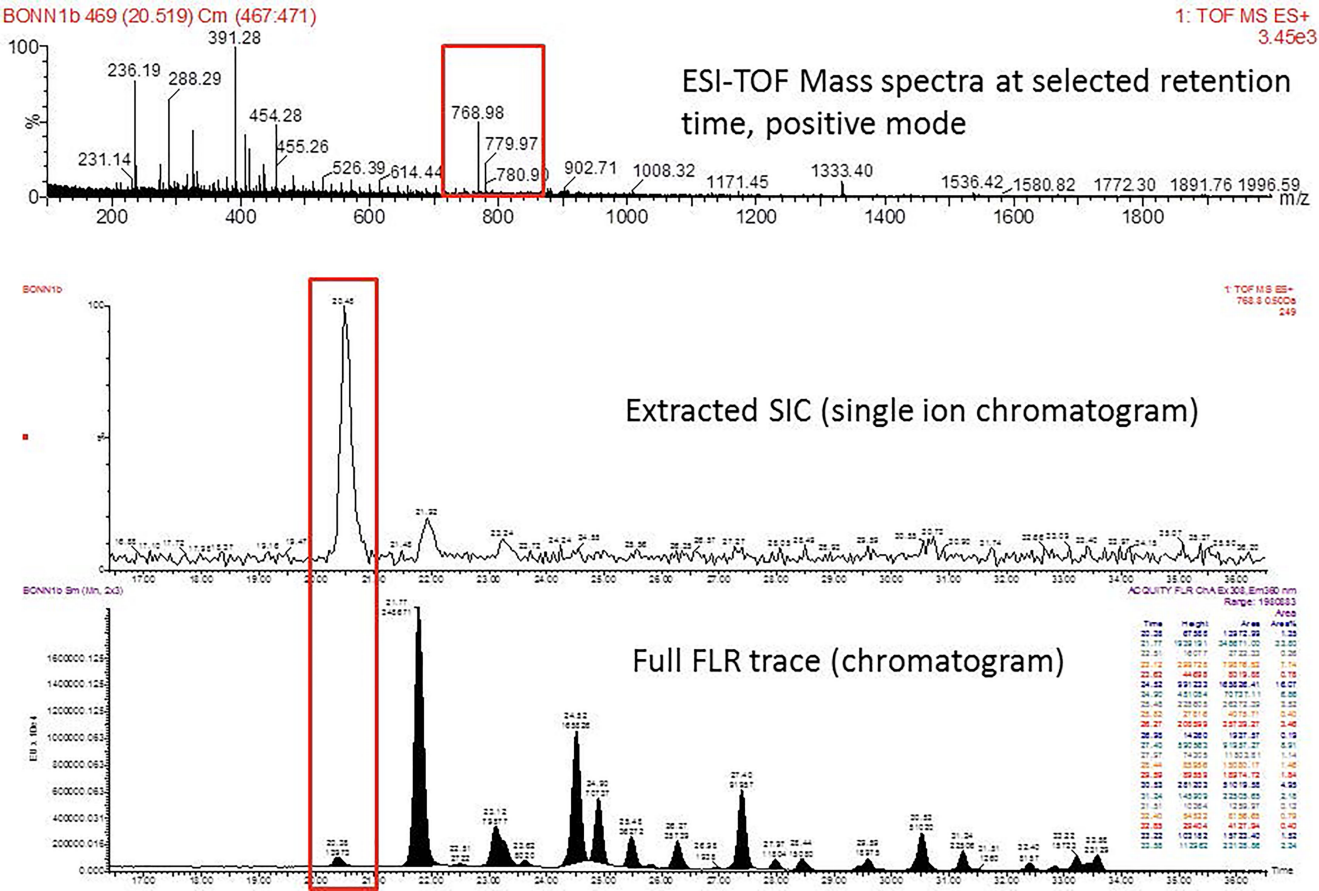
Peak assignment strategy. Profiles of procainamide-labeled fluorescent N-glycans from isolated IgG samples were obtained by HILIC UPLC-FLD/ESI-MS. Peaks were assigned according to the retention times. The graph displays a representative ESI-TOF mass spectra, a peak selection at 768.8 m/z (upper panel), the corresponding extracted Single Ion chromatogram (SIC), and full FLR trace chromatogram (middle and lower panel).

Mass distribution spectrum assigned to the glycan; ESI-MS spectrum confirming the mass found; and the fluorescence chromatogram of the sample, containing all peaks, with their corresponding retention times, total integration, and relative abundance. As an example, **Figure 2** shows the chromatogram of sample 30 for G0 glycan with a retention time of 20.9 and ESI+ mass 768.8.

### Statistical Analysis

All statistical analyses were performed using Prism 5.03 software (GraphPad Software, Inc., La Jolla, USA). Comparative analyses among groups were conducted using the Kruskal-Wallis test with a Dunn’s nonparametric posthoc test (> 2 groups). Significance was accepted when p < 0.05.

## Results

### Characterization of Glycans of IgG

We detected a total 19 glycans released from IgG purified from all tested samples. **Table 2** summarizes the proposed structures for all 19 glycans and the code used. The identified IgG-glycoforms can be separated according to the presence or not of a galactose residue. 3 glycans were agalactosylated (G0). 6 were monogalactosylated (G1), 8 glycans digalactosylated (G2) and 13 were fucosylated (F), and 6 were afucosylated. 4 glycans were monosialylated (A1), 3 glycans were disialylated (A2), whereas 12 were asialylated. 7 glycans had a bisecting GlcNAc arm (bG0F, bG1Fa, bG1Fb, bG2, bG2F, bG2A1F, bA2F).

**Table 2:** Detected glycan structures.

N-glycan	Structure	Ret. Time	ESI +
GO	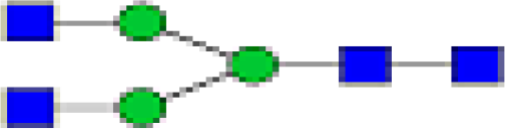	20.9	768.8
GOF	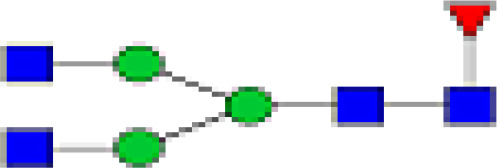	22.29	841.8
bGOF	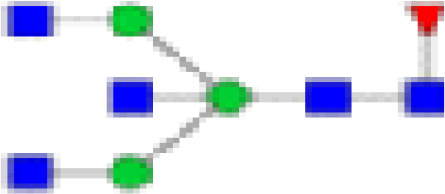	23.65	943.4
G1	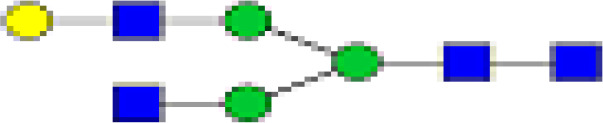	24.1	849.8
G1Fa	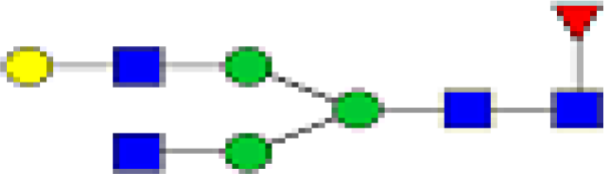	25.05	922.9
G1Fb	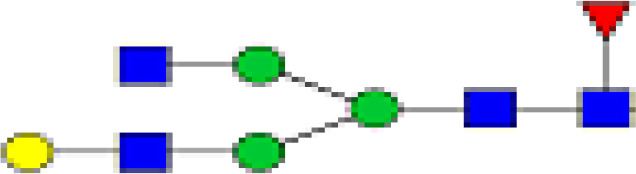	25.44	922.9
bG1Fa	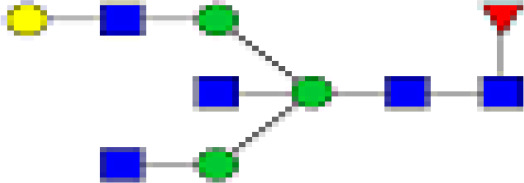	26.02	1024.4
bG1Fb	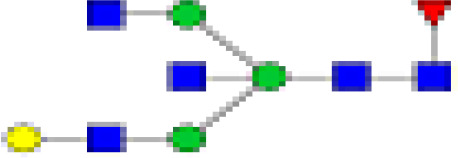	26.41	1024.4
G2	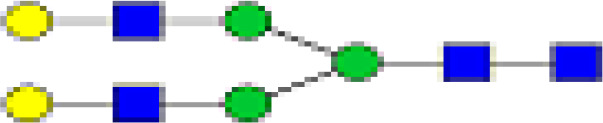	26.82	930.9
G2F	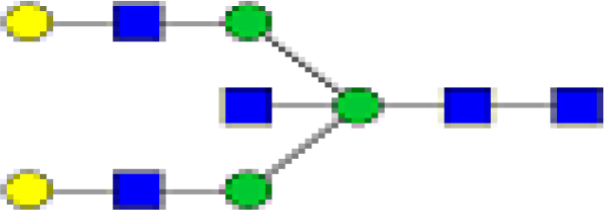	27.11	1032.5
bG2	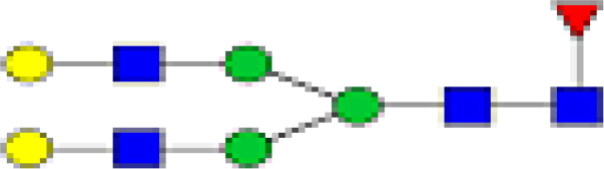	27.95	1003.8
bG2F	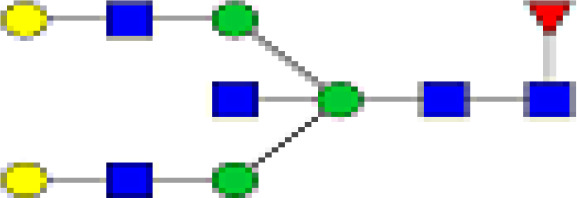	28.54	1105
G1A1F	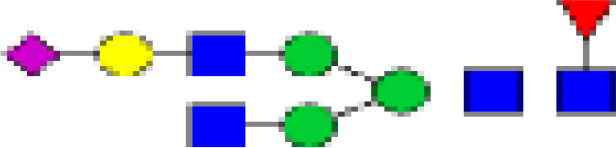	29.05	1068.5
G2A1	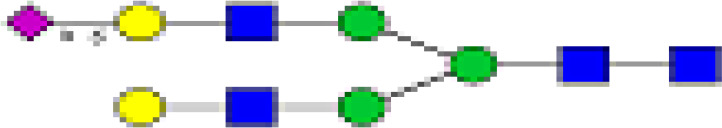	30.18	1076.5
G2A1F	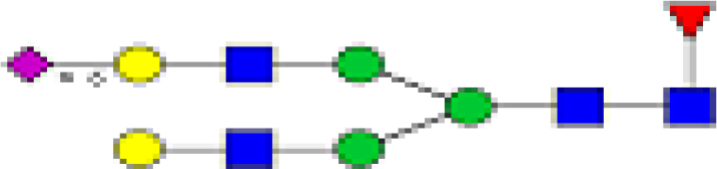	31.12	1149
bG2A1f	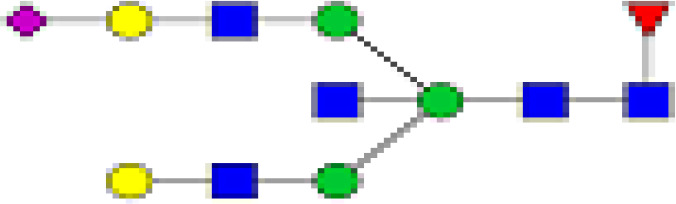	31.84	1251
G2A2	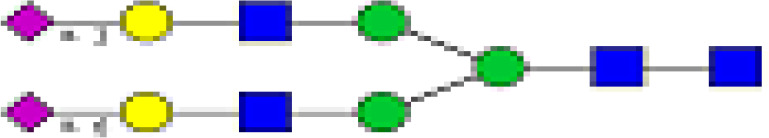	32.56	1222
A2F	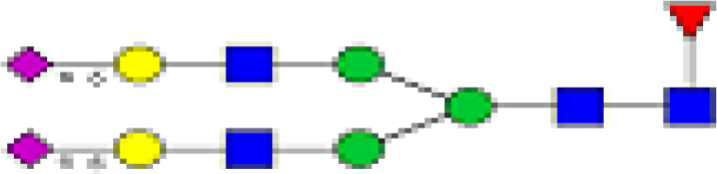	33.85	1295
bA2F	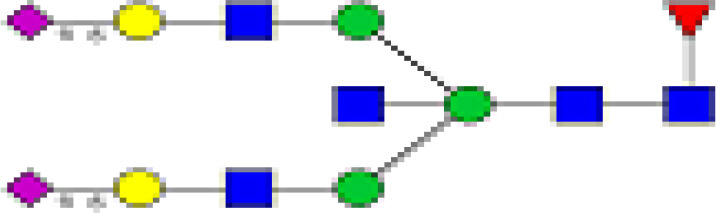	34.23	1396.6

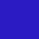
GlcNac, 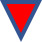
Fucose (F), 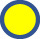 Galactose (G), 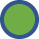 Mannose, 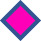 sialic acid (A).

19 different glycan structures were detected in all tested IgG samples. 3 were agalactosylated (G0), 6 were monogalactosylated (G1), 8 were digalactosylated (G2). 13 were fucosylated (F), and 6 afucosylated. 4 glycans were monosialylated (A1), 3 glycans were disialylated (A2) whereas 12 were asialyated. 7 glycans had a bisecting GlcNac arms (b...).

### Higher Heterogeneity in Antibody Glycosylation Patterns in Chronic Pathology Patients

**Figure 3:**
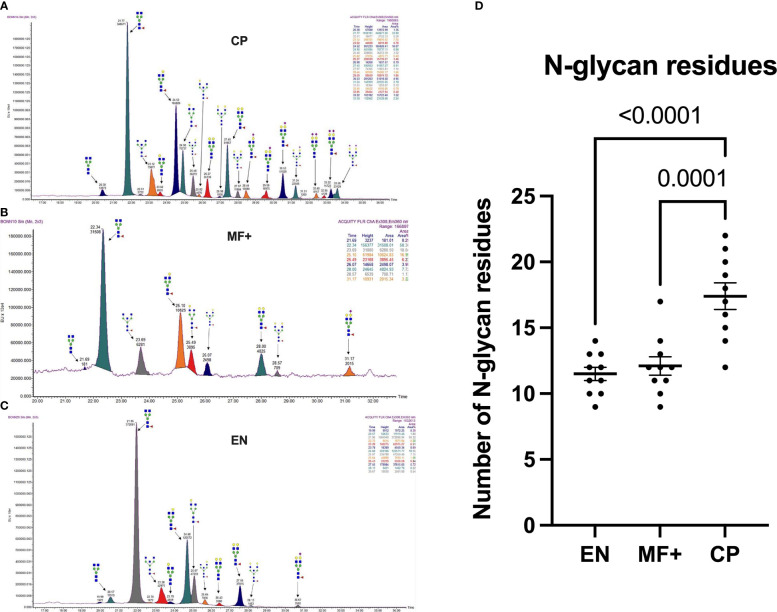
Higher IgG-Glycan heterogeneity in CP patients. **(A–C)** Representative electropherograms from a CP **(A)**, MF+ **(B)**, and EN **(C)** indicating all structures present in the Fc region of IgG. **(D)** Relative abundance of IgG molecules carrying different glycan residues in total IgG purified from EN, MF+, and CP patients. Dots represent individual donors. Bars represent means ± SEM. The indicated p-values refer to the significance level among all groups according to Kruskal-Wallis test. Indicated p values reflect the level of differences after Dunn’s multiple comparisons test. Significance is accepted when P < 0.05.

To determine whether the level of complexity in glycosylation patterns of total IgG differed between EN, CP, and MF+ donors, N-linked glycosylation patterns of total IgG were compared between the different groups. The data suggest that CP patients presented significantly more glycan residues compared to MF+ and EN. No significant difference was seen between EN and MF+ individuals regarding the number of IgG-N-linked glycan residues (**Figure 3**). This higher number of glycan residues may be linked with a more pronounced inflammatory state observed in CP patients.

### Agalactosylation Correlates With Putative Immunity

**Figure 4:**
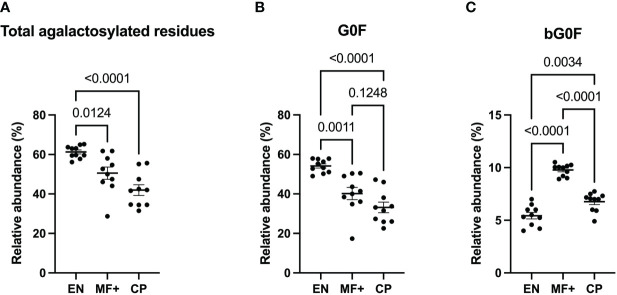
Higher levels of agalactosylated residues in EN. Graphs represent the relative abundance of all IgG-bound glycan structures lacking galactose molecules **(A)**, agalactosylated and fucosylated **(B)** or agalactosylated, fucosylated with a bisecting GlcNAc residue **(C)** in IgG purified from EN, MF+, and CP patients. Dots represent individual donors. Bars represent means SEM. The indicated p-values refer to the significance level among all groups according to the Kruskal-Wallis test and Dunn’s multiple comparisons test. Significance is accepted when P < 0.05.

Fc-galactosylation is known to improve binding to C1q in human IgG without affecting antigen-binding affinities (24). To determine the importance of IgG-N-linked galactose residues in the physiopathology of lymphatic filariasis, we analyzed the importance of lack of galactose residue on IgG of EN, MF+, and CP. Our data indicate that IgG from EN presented the highest numbers of agalactosylated residues (**Figure 4A**). This trend is confirmed when the most abundant agalactosylated residue G0F is considered (**Figure 4B**). However, the second most abundant agalactosylated residue (bG0F) was predominant in IgG of MF+ individuals (**Figure 4C**). No substantial difference was seen when considering other agalactosylated residues.

### Galactosylation Correlates With Chronic Pathology (CP)

**Figure 5:**
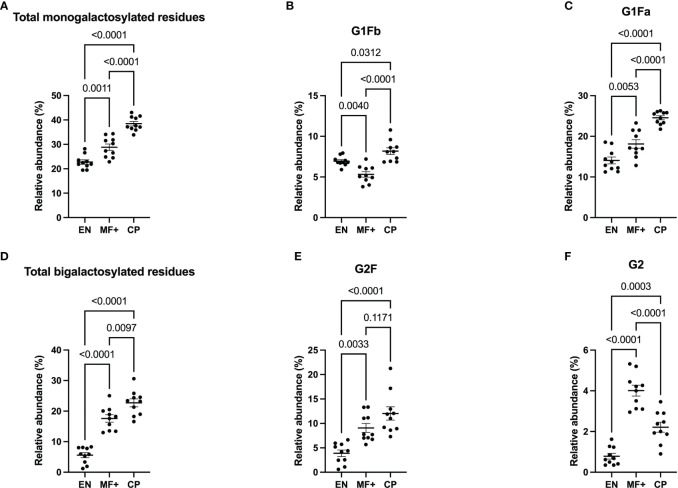
Elevated IgG- galactosylation correlates with chronic pathology. Graphs represent the relative abundance of IgG-bound glycan structures with one **(A–C)** or two galactose molecules **(D–F)** in IgG purified from EN, MF+, and CP patients. Dots represent individual donors. Bars represent means ± SEM. Statistical analyses were performed using the Kruskal–Wallis nonparametric test with Dunn’s multiple comparison post-test. Significance is accepted when *P* < 0.05.

Next, we analyzed the importance of IgG-N-linked mono and bigalactosylated residues in the physiopathology of lymphatic filariasis. The relative abundance of mono (G1, G1Fa, G1Fb, bG1Fb, G1A1F) and bigalactosylated (G2, bG2, G2F, bG2F, G2A1, G2A1F, G2A2) residues was therefore investigated on IgG of EN, MF+, and CP. The results indicate that IgG from CP patients presented the highest levels of both mono- and bigalactosylated residues. This trend was confirmed when the 3 most abundant bi and monogalactosylated residues, G2F, G1Fa, G1Fb are considered (**Figures 5B, E**). MF+ individuals generally expressed significantly higher levels of galactosylated residues compared to EN (**Figures 5A, D**), suggesting that galactosylation of IgG is primarily associated with the infection. However, IgG from MF+ individuals expressed slightly lower levels of G1Fb, a less abundant glycomer of G1Fa, compared to EN. In contrast, the afucosylated and asialylated, bigalactosylated residue G2 was found to be more abundant of IgG of MF+ individuals compared to EN and CP.

### Lower Level of Afucosylation in IgG of Asymptomatic Microfilaremic (MF+)

**Figure 6:**
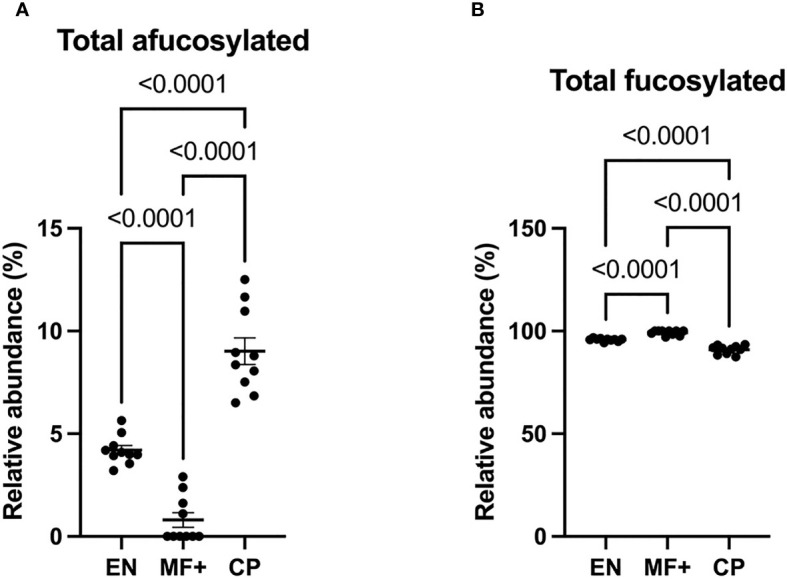
Higher levels of afucosylated IgG are associated with CP. Graphs represent the relative abundance of IgG-bound glycan structures without **(A)** or with **(B)** fucose residues **(A)** in total IgG purified from EN, MF+, and CP patients. Dots represent individual donors. Bars represent means ± SEM. Statistical analyses were performed using the Kruskal–Wallis nonparametric test with Dunn’s multiple comparison post-test. Significance is accepted when *P* < 0.05.

IgG fucosylation in the Fc domain has been attributed anti-inflammatory properties. To investigate the role of IgG-fucosylation in the physiopathology of lymphatic filariasis, we analyzed the relative abundance of fucose residues in EN, MF+, and CP individuals. Interestingly, even though high levels of fucosylated glycans were found on IgG of all groups, significantly lower amounts of afucosylated residues (and higher amounts of fucosylated residues) were found in MF+ individuals. (**Figure 6**).

### Higher Level of Sialylated Residues in IgG of Asymptomatic Microfilaremic Individuals

**Figure 7:**
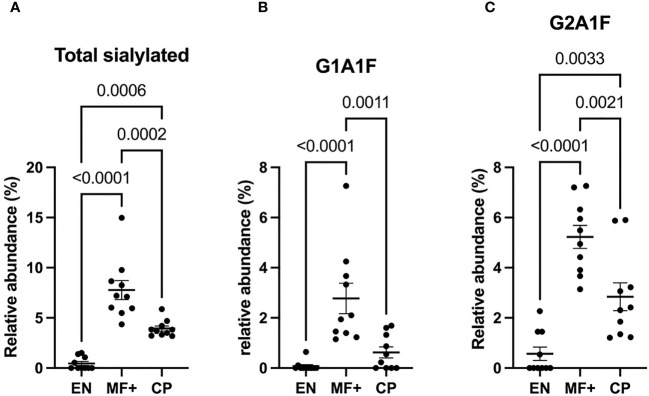
Higher levels of IgG-sialylation are associated with asymptomatic infection during LF. Graphs represent the relative abundance of IgG-bound glycan structures with sialic acid residues in total IgG purified from EN, MF+, and CP patients **(A–C)**. Dots represent individual donors. Bars represent means ± SEM. Statistical analyses were performed using the Kruskal–Wallis nonparametric test with Dunn’s multiple comparison post-test. Significance is accepted when *P* < 0.05.

We next analyzed the expression of IgG-N-linked sialic acid residues on IgG of EN, MF+, and CP. Interestingly, MF+ individuals expressed the highest amounts of sialic acid-containing residues. This increased amount is mainly due to abundant residues like G1A1F, G2A1, G2A1F, bG2A1F. All these residues are galactosylated, and apart of G2A1, are fucosylated (**Figure 7**).

### Bisecting GlcNac Is Predominant in Asymptomatic Microfilaremic Individuals (MF+)

**Figure 8:**
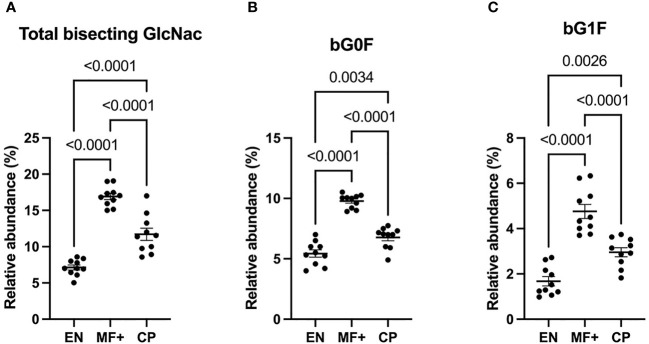
Higher levels of N-linked GlcNac are associated with asymptomatic infection during LF. Graphs represent the relative abundance of IgG bound glycan structures with bisecting GlcNac, total **(A)**, or in combination with fucose **(B)** or a galactose and fucose molecule **(C)**. Dots represent individual donors. Bars represent means ± SEM. Statistical analyses were performed using the Kruskal–Wallis nonparametric test with Dunn’s multiple comparison post-test. Significance is accepted when *P* < 0.05.

Attachment of bisecting GlcNac to the Fc glycan was shown to play a critical role in antibody-dependent cell-mediated cytotoxicity (ADCC). To determine the role of IgG-N-linked bisecting GlcNac residues in the physiopathology of lymphatic filariasis, we analyzed the presence of bisecting GlcNac residues on IgG of EN, MF+, and CP. Surprisingly, MF+ patients expressed the highest amounts of bisecting GlcNac containing residues (**Figure 8**). This predominance is mainly due to the most abundant residue with bisecting arm bG0F. However, the same trend is observed when bG1F, a monogalactosylated residue with the same core structure, is considered (**Figure 8C**).

## Discussion

The composition of the Fc N-glycan modulates the magnitude of antibodies’ effector functions, such as the antibody-dependent cell-mediated cytotoxicity (ADCC) and the complement-dependent cytotoxicity (CDC) (9). Our data suggest a higher expression in antibody glycosylation in CP patients compared to the EN and MF+ individuals. This higher level of glycosylation might be associated with the well-known pronounced inflammatory state in CP patients (19). This finding is in line with previous investigations reporting an association between the levels of Fc and Fab immunoglobulin-G-related glycosylation with inflammation (25, 26).

The present study also demonstrates that IgG-galactosylation patterns significantly differ from one lymphatic filariasis clinical group to the other. Indeed, IgG-agalactosylation correlates with putative immunity, whereas galactosylation was associated with chronic pathology. These data are in line with those obtained in a canine filaria infection model, where IgG N-galactosylation was increased as a result of infection with *Dirofilaria immitis*, a filarial nematode closely related to *Onchocerca volvulus*, *Wuchereria bancrofti*, and *Brugia malayi* (27). Even though our results converge with part of the literature, they differ from those obtained by O’Regan et al. in India, who demonstrated that IgG N-galactosylation was similar in EN, CP, and asymptomatically infected donors (28). This divergence might be associated with different factors, including the nature of the pathogens and the study population. Indeed, LF is caused in Africa mainly by *Wuchereria bancrofti*, whereas *Brugia Malayi* is the causative agent in India, Malaysia, and other parts of Southeast Asia (29). Whether these two main LF parasites have different impacts on antibody galactosylation remains to be clarified. Nevertheless, additional investigations with larger and multiregional cohorts are required to precisely define the relationship between galactosylation and the physiopathology of helminth infections. In addition, it is well established that large variability in IgG glycome exists across multiple geographical locations (30).

Higher IgG-galactosylation linked with inflammation was also seen in patients with ankylosing spondylitis, a rare form of arthritis that primarily affects the spine. In this model, the ratio of galactosylated IgG was seen to be twice higher in patients than in controls (31). The presence of terminal galactose on recombinant monoclonal antibodies was reported to enhance their capacity to induce complement-dependent cytotoxicity (CDC) by affecting their affinity to C1q (32). CDC being a key mechanism associated with the killing of microfilaria (33), the predominance of galactosylated IgG molecules in CP may explain the absence of microfilaria in this clinical group.

Regarding fucosylation, our data suggest that fucosylation was high in all clinical groups. However, when considering the relative abundance of afucosylated residues, CP patients expressed significantly higher levels of afucosylated IgG compared to both EN and MF+. Interestingly, MF+ individuals, known to have a more pronounced regulatory immune profile, presented the lowest levels of afucosylated IgG compared to EN and CP. While little data exist on IgG fucosylation in filarial infections, similar results were seen in severe acute respiratory syndrome coronavirus 2 (SARS-CoV-2) infections, where recent investigations suggest that afucosylated IgG responses promote the exacerbation of COVID-19 (34, 35). Hyperreactive immune responses being the hallmark of CP, our data confirm that excessive immune reactivity in both helminth and viral infections might be associated with elevated amounts of afucosylated IgG.

Our results also showed a significantly higher expression of sialylation in total IgG of MF+ individuals. Sialylated IgG Fc domains were shown to have the ability to increase the activation threshold of innate effector cells to immune complexes by stimulating the upregulation of the inhibitory Fc-gamma-RIIb (36, 37). MF+ individuals being well known to have more pronounced anti-inflammatory profiles, the higher expression of sialylated-IgG fits with the immune-regulated milieu in MF+ individuals (5). Our data confirmed previous findings suggesting that total IgG sialylation may be involved in asymptomatic infection during bancroftian filariasis (28).

When it comes to bisecting GlcNac, our data suggest that this feature is more pronounced in MF+ individuals compared to EN and CP. The impact of bisecting GlcNac on antibody functions is not well documented. Nonetheless, the combination of bisecting GlcNac on G0 was seen to enhance antibodies’ capacity to induce ADCC (38). ADCC being a proinflammatory process, the higher expression of IgG with bisecting GlcNac in MF+ individuals seems on the first sight to be controversial. However, the most abundant IgG glycoforms with bisecting GlcNac in MF+ individuals in our settings were also fucosylated. These findings suggest that IgG-fucosylation may interfere with the proinflammatory properties conferred by bisecting GlcNac in MF+ individuals. Indeed, branching fucose residues at the core of the biantennary glycan structure was shown to inhibit the binding affinity of the IgG-Fc to the proinflammatory receptor Fc-gamma-RIIIa (10), known to play a key role in the induction of ADCC (39).

Our data highlighted the link between immune reactivity in lymphatic filariasis and IgG glycosylation. However, the main limitation of the present study is the fact that investigations were done at total IgG level. Indeed, the glycan-mediated fine-tuning of IgG functions at subclasses and antigen-specific levels may have a more direct impact on clinical outcomes of the infection. In addition, since Fc and Fab fragments were not purified in our settings, our results encompass glycosylation patterns of both Fc and Fab fragments. It can therefore not be excluded that Fab and Fc glycosylation have distinct effects on the physiopathology of lymphatic filariasis. Recent data suggest that Fab glycan patterns are variable and play key roles in inflammation and immune regulation (26, 40). The present study focuses solely on N-glycosylation. However, in addition to N-glycosylation, O-glycosylation is also known to impact antibody effector functions (41). Given the delicate balance of protective versus pathological IgG glycosylation profiles across infectious diseases, further investigations are required to better define the implication of antibody Fab and Fc N and O-glycosylation profiles in disease outcomes. Technical limitations might also influence the results. Indeed, lower intensities were observed in some of the chromatograms. This is likely associated with the intrinsic quality of the samples. In addition, the amounts of protein obtained after protein G purification of sample 6 (EN) and 13 (MF+) were much lower compared to the other samples. For these two samples, 25 and 150 µg of protein were used respectively for deglycosylation and glycan isolation, whereas 200µg was used for all other samples. Nonetheless, excluding these two samples would have no impact on the overall outcome.

Ultimately, the present study has identified clear differences in IgG glycan profiles between CP, MF+ individuals, and EN and suggests that IgG glycome alterations might potentially be useful as biomarkers for disease severity prediction in lymphatic filariasis. A better understanding of the impact of antibody glycosylation may also provide critical insights for next-generation therapeutic formulations and vaccines.

## Data Availability Statement

The original contributions presented in the study are included in the article/supplementary material. Further inquiries can be directed to the corresponding author.

## Ethics Statement

The studies involving human participants were reviewed and approved by Ethics Committee of the University Hospital Bonn, University of Bonn, Germany. Committee on Human Research Publication and Ethics, University of Science and Technology in Kumasi, Ghana. The patients/participants provided their written informed consent to participate in this study.

## Author Contributions

All authors contributed to the article and approved the submitted version.

## Funding

This study was supported by the German Research Foundation (DFG): Project number 392112800, reference: HO 2009/13-1. AH is funded by the Deutsche Forschungsgemeinschaft (DFG, German Research Foundation) under Germany’s Excellence Strategy – EXC2151 – 390873048”.

## Conflict of Interest

The authors declare that the research was conducted in the absence of any commercial or financial relationships that could be construed as a potential conflict of interest.

## Publisher’s Note

All claims expressed in this article are solely those of the authors and do not necessarily represent those of their affiliated organizations, or those of the publisher, the editors and the reviewers. Any product that may be evaluated in this article, or claim that may be made by its manufacturer, is not guaranteed or endorsed by the publisher.

## Abbreviations

EN, endemic normals; MF, microfilariae; CP, chronic pathology; IgG, immunoglobulin G; GlcNac, N-Acetylglucosamine; CFA, circulating microfilaria antigen; UPLC-FLD/ESI-MS, ultra-performance liquid chromatography-fluorescence detector/electrospray ionization-mass spectrometry.
